# *WONKA* and *OOMMPPAA*: analysis of protein–ligand interaction data to direct structure-based drug design

**DOI:** 10.1107/S2059798316009529

**Published:** 2017-02-24

**Authors:** Charlotte M. Deane, Ian D. Wall, Darren V. S. Green, Brian D. Marsden, Anthony R. Bradley

**Affiliations:** a Oxford Protein Informatics Group, Department of Statistics, University of Oxford, 24–29 St Giles, Oxford OX1 3LB, England; b Computational and Structural Chemistry, GlaxoSmithKline Medicines Research Centre, Gunnels Wood Road, Stevenage SG1 2NY, England; c SGC, Nuffield Department of Medicine, University of Oxford, Old Road Campus Research Building, Roosevelt Drive, Headington, Oxford OX3 7DQ, England; d Kennedy Institute of Rheumatology, Nuffield Department of Orthopaedics, Rheumatology and Musculoskeletal Sciences, University of Oxford, Roosevelt Drive, Headington, Oxford OX3 7FY, England

**Keywords:** computational chemistry, structure-based drug design, *WONKA*, *OOMMPPAA*

## Abstract

In this work, two freely available web-based interactive computational tools that facilitate the analysis and interpretation of protein–ligand interaction data are described. Firstly, *WONKA*, which assists in uncovering interesting and unusual features (for example residue motions) within ensembles of protein–ligand structures and enables the facile sharing of observations between scientists. Secondly, *OOMMPPAA*, which incorporates protein–ligand activity data with protein–ligand structural data using three-dimensional matched molecular pairs. *OOMMPPAA* highlights nuanced structure–activity relationships (SAR) and summarizes available protein–ligand activity data in the protein context. In this paper, the background that led to the development of both tools is described. Their implementation is outlined and their utility using in-house Structural Genomics Consortium (SGC) data sets and openly available data from the PDB and ChEMBL is described. Both tools are freely available to use and download at http://wonka.sgc.ox.ac.uk/WONKA/ and http://oommppaa.sgc.ox.ac.uk/OOMMPPAA/.

## Abstract 2

The background to and development of *WONKA* and *OOMMPPAA*, tools for structure-based drug design, are described.

## 1. Introduction   

Technological advances in high-throughput crystallography and protein–ligand biophysical and biochemical binding assays have resulted in a rapid increase in the quantity of liganded crystal structures and high-quality activity data points for many protein targets (Badger, 2012 ▸; Zheng *et al.*, 2014 ▸). Concerted efforts to consolidate and store such data have generated large and highly curated data sets both in the private (for example corporate databases) and public domains (Berman *et al.*, 2003 ▸; Gaulton *et al.*, 2012 ▸). Further, it is now commonplace for an industry structure-based drug-design (SBDD) programme to have access to many tens of liganded crystal structures and many thousands of high-quality activity data points.

**Figure 1:**
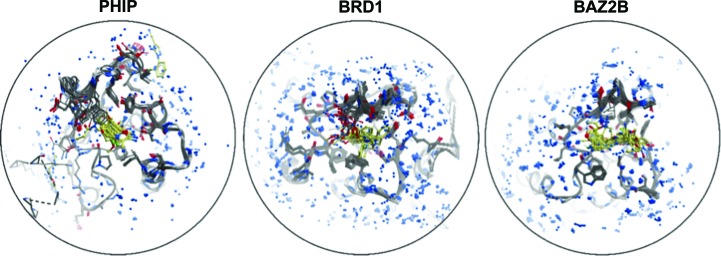
The ensemble of liganded structures for human PHIP, BRD1 and BAZ2B bromodomains, respectively (left to right). The superimposition of structures results in a visualization which is extremely difficult to interpret, especially when attempting to identify nuanced changes present in a minority of structures. The proteins are shown as grey sticks, the ligands are shown as yellow sticks and waters are shown as blue spheres.

At the same time, computational tools have not kept pace with this influx of data. Analysis of the output of structural ensembles is often carried out with tools such as *PyMOL* (Schrödinger), which was designed for the evaluation of at most a handful of structures at once. Such tools do not naturally identify and highlight the core trends within a data set. Furthermore, they do not lend themselves to the capture and sharing of important observations from such ensembles. Fig. 1 ▸ shows three examples of such ensembles for three human bromodomain targets. Much of the important information in each ensemble is obfuscated by the sheer quantity of data available, which current software platforms are unable to untangle in an intuitive and accessible manner. It is therefore timely to create tools which consider all information from such large data sets in a holistic and unbiased manner.

Analysis of activity data within an SBDD programme is also challenging. One of the most widely used methods for the analysis of such activity data is three-dimensional quantitative structure–activity relationships (3D QSAR; Verma *et al.*, 2010 ▸). In 3D QSAR, statistical models are generated to relate small-molecule bioactivity data to three-dimensional compound properties. However, there are several well known problems with 3D QSAR (Scior *et al.*, 2009 ▸). Firstly, generating appropriate three-dimensional conformations, in particular when dealing with varied binding modes, is highly challenging. Secondly, if one uses simple methods (*e.g.* linear regression models) they are unable to find nuanced features in activity data. Finally, if more elaborate methods are used (*e.g.* machine learning) they require careful expert implementation, can be prone to overfitting (Hawkins, 2004 ▸) and can be difficult to interpret (Cherkasov *et al.*, 2014 ▸).

**Figure 2:**
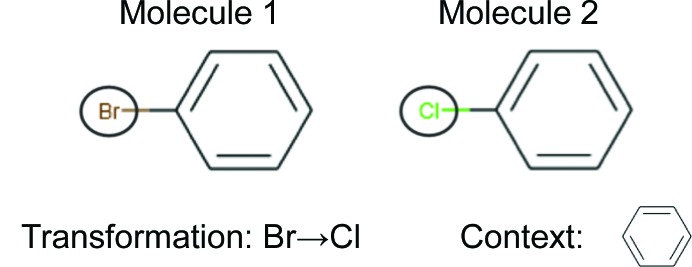
Example of a matched molecular pair (MMP). Molecule 1 and molecule 2 form a matched molecular pair. Bromine to chlorine is the transformation and the context is a phenyl ring.

Analysing activity data in a pairwise manner can circumvent some of the pitfalls and generalizations of 3D QSAR. A method to carry out such pairwise analysis is matched molecular pair analysis (MMPA; Dossetter *et al.*, 2013 ▸). As shown in Fig. 2 ▸, a matched molecular pair (MMP) consists of two compounds that are identical apart from one small structural alteration, known as a transformation (Hussain & Rea, 2010 ▸). From analysing the aggregate effects of such transformations over multiple different series, one can then assess the impact of a specific transformation upon a given compound property (*e.g.* protein–ligand binding affinity).

The example MMP shown in Fig. 2 ▸ is a two-dimensional MMP (2D MMP), since three-dimensional coordinate information is not provided. 2D MMP approaches have been shown to be effective for a wide range of properties (Papadatos *et al.*, 2010 ▸). A natural extension of 2D MMPPA is to include three-dimensional structural information from structure ensembles in the analysis (3D MMPA). A central advantage of 3D MMPA is that structure–activity relationships (SAR) can be projected between structurally and pharmacophorically dissimilar series that have analogous binding modes (Posy *et al.*, 2013 ▸). Critically, 3D MMPA enables analyses of transformations within the local residue environments, since this environment will directly affect the impact of a given transformation. 3D MMPA also presents a number of key advantages over 3D QSAR. Firstly, it provides a simple and reliable route to three-dimensional conformation generation (Klei *et al.*, 2014 ▸; Posy *et al.*, 2013 ▸). Secondly, it generates models that are related to individual and assessable pairwise comparisons. From this, nuanced trends (Bradley *et al.*, 2014 ▸) and confounding factors in data can be readily observed.

The above challenges and developments in the analysis of protein–ligand interaction data led to the development of the *WONKA* and *OOMMPPAA* methods. *WONKA* is an analysis tool for ensembles of protein–ligand structures, providing a simple interactive tool to find trends within a set of structures of the same protein. *WONKA* presents analyses of water displacements, residue movements, ligand-binding sites and ligand-based pharmacophores. These are then related to the individual ligands in an ensemble. They, for example, allow the user to quickly and easily determine which ligand or ligands displace a given conserved water molecule. *OOMMPPAA* extends upon *WONKA* by using a 3D MMPA approach to incorporate available activity data into the context of known structural data. From this, the distribution and nature of the available SAR can be analysed in the context of the protein binding site. *OOMMPPAA* uses pharmacophore-based abstractions to then highlight concerted effects across multiple ligands. Both tools are freely available to download and try online.

In the following sections, we outline the two methods and demonstrate their application to SGC data and data from the Protein Data Bank (PDB) and ChEMBL databases.

## 2. Methods and materials   

A full description of both the *WONKA* and *OOMMPPAA* methods can be found in separate publications [Bradley *et al.* (2015) ▸ and Bradley *et al.* (2014) ▸, respectively]. Data are stored in a bespoke Python Django (Django Software Foundation, 2013 ▸) data model that is common to both applications. All computational chemistry processing was carried out using *RDKit* (Landrum; http://www.rdkit.org). The input data for both tools are pre-aligned PDB files of protein–ligand complexes. A comma-separated variable (CSV) file is required to indicate the path to the PDB file, and the SMILES (Weininger, 1988 ▸) specification is required for the ligand bound to that protein. Additionally, for *OOMMPPAA* activity data are required and are input as a separate CSV file. Here, we give a brief overview of the methods.

*WONKA* processes its input data in four steps. Firstly, the PDB files are parsed and the ligands are extracted. Secondly, fragments and pharmacophores are generated from the bound ligands. Thirdly, waters, residues, ligand pharmacophores, ligand fragments and ligands are clustered in space. Finally, these clusters are taken from the data model and displayed in an interactive web-browser-based application. The *OOMMPPAA* processing method consists of four further steps that build upon the data processed by *WONKA*. Firstly, the matched molecular pair database is formed using the method of Hussain and Rea and the fragments generated from *WONKA* (Hussain & Rea, 2010 ▸). Secondly, all matched molecular pairs are found where one compound of each pair is represented in a crystal structure and the other is not. The compound with the crystal structure in each pair is used to predict the coordinates of compounds for which no crystal structure is available. Thirdly, pharmacophore differences between compounds in each 3D MMP are found. Finally, the differences found in this last step are displayed and can be queried in the three-dimensional interactive viewer.

### 2.1. Input data   

In the following analysis, the input data for *WONKA* are taken from the SGC database and are for the second bromodomain of human pleckstrin homology domain-interacting protein (PHIP; UniProt accession Q8WWQ0). The *OOMMPPAA* analysis is of human carbonic anhydrase 2 (UniProt accession P00918) and the first and second bromo­domain of human bromodomain-containing protein 4 (BRD4; UniProt accession O60885). The input activity data were taken from ChEMBL v.19. The data were then filtered to only allow *in vitro* IC_50_ and *K*_i_ data with a ChEMBL confidence score of 7 or greater. Structural data were derived from the PDB, in which all ligand-bound structures were found. The lowest resolution was 2.8 Å; however, the majority of the structures were at better than 2.0 Å resolution. The BRD4 data set was supplemented by 14 internal SGC protein–ligand structures. Carbonic anhydrase and BRD4 data were chosen as they exemplify the utility of *OOMMPPAA* with large (carbonic anhydrase 2; 4140 activity data points and 286 liganded co-crystal structures) and small (BRD4; 265 activity data points and 90 liganded co-crystal structures) data sets. Protein–ligand structures were aligned using Molsoft *ICM* sequence-based alignment. The input data are summarized in Table 1 ▸.

### 2.2. *WONKA* analysis page   

**Figure 3:**
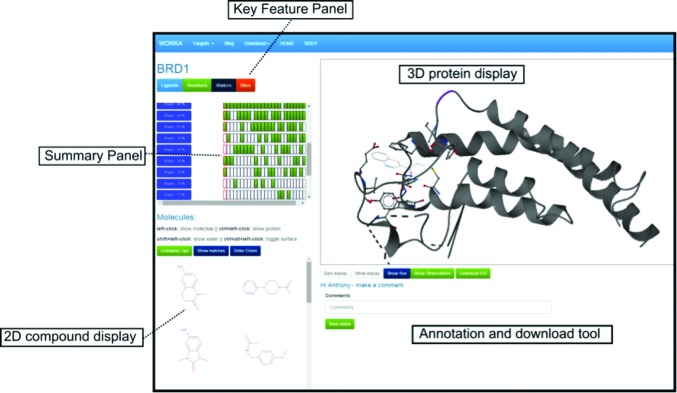
A screenshot of the *WONKA* analysis page for PHIP. The Key Feature panel has four buttons: Ligands, Residues, Waters and Sites. Clicking on each button allows the user to show different analysis in the Summary Panel. In this case the Water analysis is shown. The two-dimensional compound display allows the user to view the available ligands for this target. Clicking on each compound in the two-dimensional display shows the three-dimensional conformation of the ligands and their parent protein/water molecules in the three-dimensional protein display. The annotation and download tool allows the user to make and share observations and download the data in ICB format.

Fig. 3 ▸ shows a screenshot of the web-based *WONKA* analysis page for PHIP. The page is made up of five main components. Firstly, the Key Feature Panel allows the user to select analyses based on Ligand, Residue, Water or Site, respectively. Clicking on each button alters the information shown in the second core component, the Summary Panel. Each row in the Summary Panel relates to a different feature, in this case conserved water molecules. Each column relates to a different ligand and is coloured green if that ligand has that conserved water (and is uncoloured if not). Thirdly, the three-dimensional protein display is a fully interactive three-dimensional visualization of the selected structural data and is powered by *ActiveIcmJS* (Raush *et al.*, 2009 ▸). Fourthly, three-dimensional ligand structures and their respective waters and proteins can be displayed or undisplayed using the two-dimensional compound display. Finally. the annotation and download tool allows the user to save and annotate interactive three-dimensional snapshots and share them with anyone in the world *via* a URL.

### 2.3. *OOMMPPAA* analysis page   

**Figure 4:**
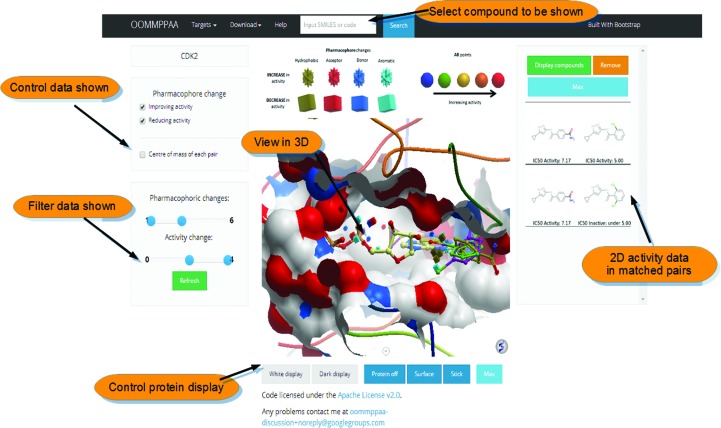
A screenshot of the interactive web-based viewer for *OOMMPPAA*. Compounds are queried using the search bar at the top. The top-left check boxes and sliders control the points shown in the three-dimensional display. The central display shows three-dimensional molecular visualizations. The right-hand bar shows two-dimensional activity data selected by the user in the three-dimensional display.

The *OOMMPPAA* method provides a separate three-dimensional visualization tool in which the activity data of a target can be assessed in the context of the protein binding site. Fig. 4 ▸ shows a screenshot of the *OOMMPPAA* web-based display, showing the analysis of PDB data and ChEMBL data for cyclin-dependent kinase 2 (CDK2). Firstly, in the top bar the ligand shown in the three-dimensional display can be changed by searching for the relevant SMILES specification (two-dimensional molecular description; Weininger, 1988 ▸). Secondly, the activity-improving or activity-reducing pharmacophore points can be displayed using the left-hand panel. As an example, one can display only hydrogen-bond acceptor pharmacophore points that are associated with a log increase in *in vitro* activity of greater than 1. Thirdly, the three-dimensional ligand and protein coordinates can be controlled and shown in the three-dimensional *ActiveIcmJS* display (Raush *et al.*, 2009 ▸). Finally, 3D MMPs can be selected and their underlying activity data can be shown in the right-hand panel.

### 2.4. Application of *OOMMPPAA* to a small data set   

**Figure 5:**
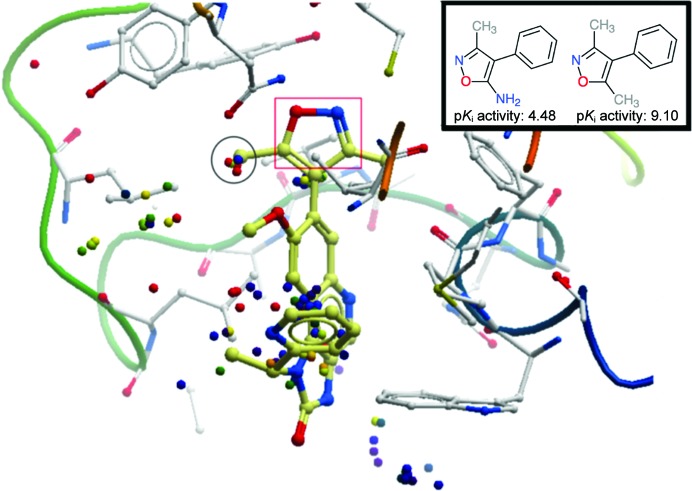
An *OOMMPPAA* view of available 3D MMP activity data for BRD4. The ligand (I-BET151) bound to BRD4 (PDB entry 3zyu) is shown as thick cream sticks and the protein as white sticks and ribbon. Each matched molecular pair is shown as a sphere coloured by the activity change associated with it from blue (low) to red (high) and positioned at the centre of mass of the nonmatching moiety of the 3D MMP. In the red box, the isoxazole motif key for binding is highlighted. In the black circle several red spheres (high activity changes) are around a putatively important methyl group. Transformations corresponding to these spheres (not shown) indicate several transformations from a methyl fragment to other substituents, all of which cause a drop in activity. One of these transformations (inset) shows a large activity change.

In this section, we outline the use of *OOMMPPAA* in analysing available structural and activity data for BRD4. For this target only limited activity data are available (265 activity data points). Fig. 5 ▸ shows the first bromodomain of BRD4 with I-BET151 bound (PDB entry 3zyu; Dawson *et al.*, 2011 ▸). Each three-dimensional matched molecular pair is shown as a sphere which is coloured based on activity change from blue (low) to red (high). The coordinates of each sphere are the centres of mass of the nonmatching moiety in the 3D MMP, *i.e.* the transformed component of the compound. In this way, the points indicate the coverage of available activity data for this protein target.

Two conclusions can be drawn from the distributions of points. Firstly, the density of points is low and the coverage of points is not uniform, as would be expected for a target with such little activity and few structural data. For example, no spheres (MMPs) can be found near the region where the isoxazole ring binds to the asparagine residue (red square in Fig. 5 ▸). This analysis would suggest that the synthesis and testing of compounds exploring this region may be instructional. Secondly, the functionally important methyl substituent (circled) is surrounded by spheres. Most of these spheres are red, indicating that many of the transformations in this region involve large activity changes. Exploration of this SAR through *OOMMPPAA* indicates that replacing a methyl group in this region with a chloro, bromo, hydroxyl or amino group reduces activity. This information would therefore discourage these transformations in the future.

As discussed in §[Sec sec1]1, the pairwise analysis in *OOMMPPAA* can be used to detect confounding factors in analysis. In the inset in Fig. 5 ▸ we show an example of such a confounding factor. The 3D MMP shown presents an activity change of over four orders of magnitude on converting an amino group to a methyl group. This is unusually large for such a minor transformation. Inspection of the data within *OOMMPPAA* shows that the fragment with a dimethylated isoxazole has subnanomolar (*K*_i_ = 0.8 n*M*, p*K*_i_ = 9.10) activity. It is highly unlikely that a fragment of only 13 heavy atoms would present such a high activity. Inspection of ChEMBL data indicates that the same fragment measured in bioactivity assays by a separate research group showed an activity of 84.2 µ*M*, which is more appropriate for such a fragment. Both activities were IC_50_ values but were from different assay types: the first (0.8 n*M*) was from a fluorescence anisotropy assay and the second (84.2 µ*M*) was from a peptide-displacement assay.

Such inconsistencies will always exist in databases, in particular when the data are collated from multiple laboratories using different assay formats, as ChEMBL is. In 3D QSAR model building, for example, such data sets must be cleansed before use. This cleansing often occurs in an automated manner resulting in, at best, the removal of these data and, at worst, a poor choice of which data points in the set to be kept. Clearly, the benefit of pairwise comparison of data and enabling the user to inspect the data themselves allows such confounding factors to be observed and acted upon, whilst not losing any available data. In the above examples, we have shown that the 3D MMPA in *OOMMPPAA* can provide useful but limited analysis for smaller data sets and can be used to find confounding factors within activity data sets.

### 2.5. Application of *OOMMPPAA* to a large data set   

**Figure 6:**
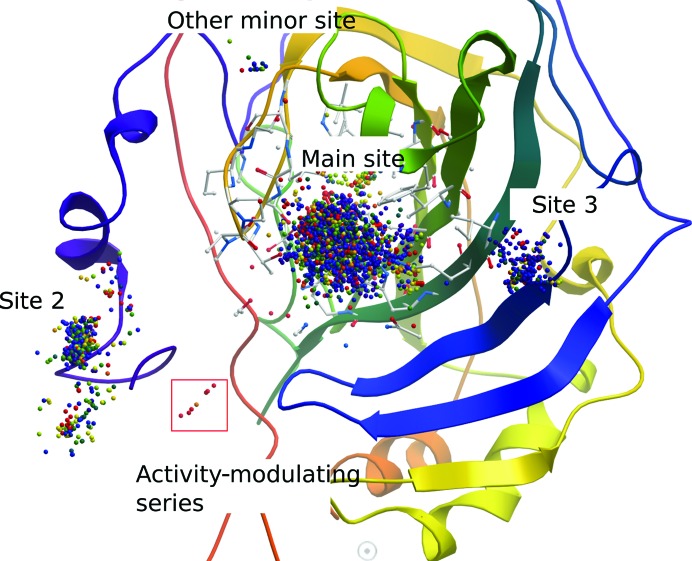
An example of analysing a larger data set. An *OOMMPPAA* view of available carbonic anhydrase 2 data. Each matched molecular pair is shown as a sphere coloured by the activity change associated with it from blue (low) to red (high). Firstly, the high data density of this larger data set can be observed by the concentration of points compared with BRD4. Secondly, different regions of binding in the carbonic anhydrase can be observed. Thirdly, one of these sites contains only red and orange spheres, indicating that the associated MMPs convey large activity changes.

*OOMMPPAA* analysis of freely available carbonic anhydrase 2 data demonstrates the power of the tool to analyse larger data sets. Fig. 6 ▸ shows the carbonic anhydrase 2 protein, with the active site shown as white sticks and the protein backbone as a ribbon. Each 3D MMP is shown as a coloured sphere (with the coordinates being the centre of mass of the transformed moiety of the MMP in each case), coloured by the activity change, from blue (low) to red (high), associated with that 3D MMP. Two core observations can be made from this figure. Firstly, a much larger density of points can be seen in Fig. 6 ▸ than in Fig. 5 ▸. Protein–ligand structures of 286 unique ligands are available for this target and 4140 unique compounds have been tested in bioactivity assays. The scale of this data set is more representative of that found in significant SBDD programmes such as those in industrial settings. Secondly, the major regions of SAR for this target can be seen in the context of the protein binding site. *OOMMPPAA* displays three major clusters (main site, site 2 and site 3) and two less populated regions of SAR (activity-modulating series, other minor site).

**Figure 7:**
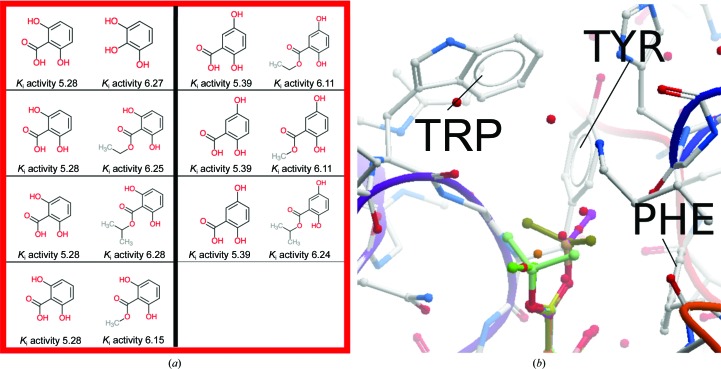
(*a*) The matched molecular pairs corresponding to these spheres indicates a series of low-molecular-weight molecules with large activity changes from very minor molecular transformations (*e.g.* adding a methyl group increases activity by over one log unit). (*b*) The three-dimensional conformations of these compounds (rainbow sticks) project into a hydrophobic pocket (tryptophan, TRP; tyrosine, TYR; phenylalanine, PHE), rationalizing the favourability of hydrophobic substituents. The protein is shown as white sticks in (*b*).

*OOMMPPAA* can then be used to highlight and then investigate interesting SAR within such large data sets. The red box in Fig. 7 ▸(*a*) highlights an ‘activity-modulating series’. The spheres in this region are coloured orange and red, meaning that they represent relatively large activity changes. In Fig. 7 ▸(*a*) we show the two-dimensional transformations and activity data for these 3D MMPs. Within this series a one log gain in activity is seen on adding a methyl, ethyl or isopropyl group, indicating that hydrophobic alkyl groups are favoured. Fig. 7 ▸(*b*) shows an *OOMMPAA* screenshot of the available 3D MMPs in these series, placing the two-dimensional SAR in Fig. 7 ▸(*a*) into the protein context. It shows that these alkyl groups project into a hydrophobic pocket of carbonic anhydrase 2 containing a phenylalanine, a tyrosine and a tryptophan residue. By combining the information from Figs. 6 ▸, 7 ▸(*a*) and 7 ▸(*b*), *OOMMPPAA* highlights an interesting series of ligands that show large activity changes. Investigation of these series then provides clear experimental evidence of the positive effect of hydrophobic residues on *in vitro* activity in this pocket of carbonic anhydrase 2. In this example, we show how *OOMMPPAA* can summarize the data for a large data set in a clear and intuitive manner and highlight interesting SAR in the context of the protein binding site.

## 3. Conclusions   

In this paper, we describe the background for and use of the *WONKA* and *OOMMPPAA* platforms. Both methods are freely available interactive computational tools designed to analyse and describe the influx of protein–ligand interaction data associated with SBDD programmes. *WONKA* is a tool to summarize large ensembles of protein–ligand structures of the same protein target. *WONKA* also provides a platform for annotation and data sharing within and between research groups, a feature that is invaluable in the context of working in a multi-disciplinary team. *OOMMPPAA* builds upon *WONKA* to incorporate available activity data in the context of the binding sites of protein–ligand structures using a 3D MMP approach. Further, we show the use of *OOMMPPAA* in interrogating available activity data for smaller (BRD4) and larger (carbonic anhydrase 2) structural and activity data sets. Both *WONKA *and *OOMMPPAAA* are freely available to try online and are free to download at http://oommppaa.sgc.ox.ac.uk/OOMMPPAA/ and http://wonka.sgc.ox.ac.uk/WONKA/.

## Figures and Tables

**Table 1:** The structural and activity data sets used in this work

Target	Co-crystal structures	Bioactivity data
PHIP	12	NA
Carbonic anhydrase 2	286	4140
BRD4	90	265
CDK2	261	1632
